# TDMQ20 as A Drug Candidate for Wilson’s Disease: Comparison with D-Penicillamine, Trientine, and Tetrathiomolybdate In Vitro and In Mice

**DOI:** 10.3390/pharmaceutics17091237

**Published:** 2025-09-22

**Authors:** Yingshan Zhu, Weiling Peng, Guangwei Liu, Longxin Li, Zikang Zhou, Michel Nguyen, Anne Robert, Yan Liu, Bernard Meunier

**Affiliations:** 1 School of Chemical Engineering and Light Industry, Guangdong University of Technology (GDUT), Higher Education Mega Center, Guangzhou 510006, China; eshan202506@outlook.com (Y.Z.); pengweiling@mails.gdut.edu.cn (W.P.); 2112306084@mail2.gdut.edu.cn (G.L.); 2112406028@mail2.gdut.edu.cn (L.L.); 2112406008@mail2.gdut.edu.cn (Z.Z.); 2 Laboratoire de Chimie de Coordination du CNRS, Inserm ERL 1289, 205 route de Narbonne, 31077 Toulouse, Cedex 4, France; michel.nguyen@lcc-toulouse.fr (M.N.); anne.robert@lcc-toulouse.fr (A.R.)

**Keywords:** copper chelator, liver, toxic milk mouse, ceruloplasmin, Wilson’s disease

## Abstract

**Background/Objectives**: The lifelong treatment of Wilson’s disease (WD) currently relies on copper chelators with relatively poor metal specificity, which frequently exhibit serious adverse effects. There is a real medical need for a specific copper chelator to regulate the copper excess efficiently, at lower doses than those used for penicillamine (DPA) or trientine (TETA), and with lower toxicity in long-term treatments. **Methods**: The efficiency of the specific Cu(II) chelator named TDMQ20 was evaluated by oral treatment of TX mice, used as a WD model, and compared with those of DPA, TETA, and also tetrathiomolybdate (bcTTM). We documented TDMQ20′s ability to (i) decrease the hepatic copper load, (ii) increase the amount and ferroxidase activity of ceruloplasmin (CP), and (iii) regulate liver proteins that are impaired in WD mice. **Results**: Compared to the other copper chelators, TDMQ20 was the only one that efficiently mediated excretion of Cu and restoration of active ceruloplasmin levels at doses 8 times lower than DPA. Such efficacy is related to the design of this chelator, which specifically coordinates Cu(II) as a discrete and soluble complex. Conversely, DPA, TETA, and bcTTM give rise to various complexes with copper ions, often with oligomeric or cluster structures that can be retained in blood circulation or sequestered by proteins. **Conclusions**: Taking into consideration all the advantages of TDMQ20 compared to other ligands, including its lack of toxicity during long-term administration in mice, the drug candidate TDMQ20 appears to be a first-class challenger to the currently used treatments, i.e., DPA, TETA, and bcTTM.

## 1. Introduction

Wilson’s disease (WD) is due to mutations of *ATP7B*, the gene coding for ATP7B, a copper-transporting ATPase regulating the copper content of human cells by transporting the cytoplasmic excess of copper to the trans-Golgi compartment for incorporation into apo-ceruloplasmin, followed by excretion out of cells. In hepatocytes, ATP7B plays a pivotal role in copper homeostasis. In the presence of an elevated Cu concentration, ATP7B redistributes from the trans-Golgi network to hepatocytes’ apical pole for transport of the excess copper into bile [1,2,3]. The deficiency of this copper carrier causes the accumulation of copper in the liver and, over time, in advanced disease, in the brain and various organs. To avoid fulminant hepatic failure and various other potentially fatal issues, lifelong treatment is therefore necessary, using copper chelators to facilitate the excretion of copper.

**Figure 1:**
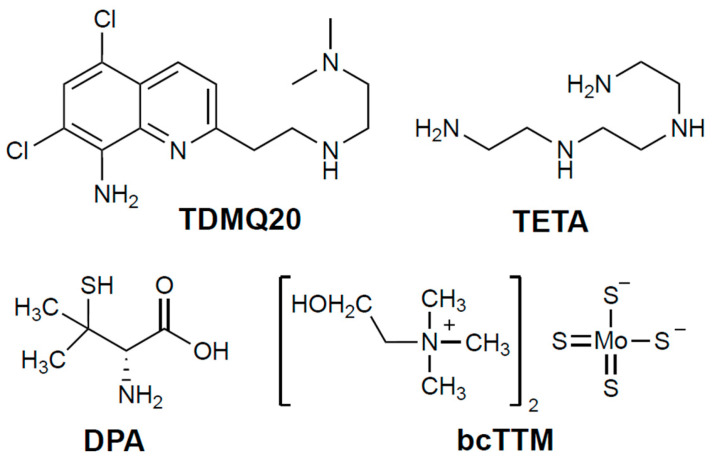
Structures of the chelators TDMQ20, DPA, TETA, and bcTTM (base forms).

The current first-line oral treatment is based on D-penicillamine (DPA, 3-mercapto-D-valine), and the second-line treatment is trientine (triethylene-tetramine, TETA) when DPA is inefficient or not tolerated (for structures, see Figure 1). In fact, both drugs are used at high doses (750–1500 mg/day of DPA or 900–2700 mg/day of TETA, for an adult), and exhibit frequent adverse effects, such as bone marrow depression, anemia, skin rash, hemorrhagic gastritis, and irreversible worsening of neurological symptoms, which may be serious enough to require discontinuation of treatment (30% of patients treated with DPA) [4,5,6]. These chelators are often associated with zinc salts in order to induce metallothionein synthesis and, consequently, to prevent copper intestinal absorption [3]. However, the limitations of treating patients at the same time with a metal (Zn) and a non-selective metal chelator have been emphasized [4].

There is thus a real medical need for a specific copper chelator to regulate the copper excess efficiently, at lower doses than those used for DPA or TETA, and with fewer side effects in the context of long-term treatments. TDMQ20 (Figure 1) is a copper(II)-specific chelator that can reverse memory loss in the early stages of a murine model of Alzheimer’s disease [7]. We recently reported that low doses of TDMQ20 (25–50 mg/kg) are more efficient at decreasing the hepatic concentration of copper and improving its physiological excretion pathway than DPA at 200 mg/kg/day. TDMQ20 also increases the serum concentration of ceruloplasmin a signature of the normal situation [8]. In addition, contrary to DPA, TDMQ20 does not inhibit the in vitro activity of copper-dependent enzymes, such as Cu,Zn-superoxide dismutase (Cu,Zn-SOD), and does not induce oxidative stress in the presence of copper ions, a feature accounting for a better safety profile than that of DPA [8].

Ammonium tetrathiomolybdate [(NH_4_)_2_(MoS_4_)] has been considered for decades as a possible alternative to DPA and TETA in the treatment of WD [9]. This idea was born from the evidence of a severe copper deficiency in grazing ruminants in several places of the world where foodstuffs naturally rich in molybdenum are consumed, despite a rather high level of copper present in pasture soil. In fact, in the sulfur-rich rumen, molybdate is converted to thiomolybdate, which readily reacts with dietary copper to yield an insoluble copper tetrathiomolybdate polymer, thus causing copper sequestration and depletion in animals [10]. Ammonium tetrathiomolybdate (Coprexa^®^) at an oral dose of 60 mg/day was proposed as a treatment for WD [4,11] but has never been approved [12]. Bis-choline-tetrathiomolybdate (WTX101, also now known as ALXN1840 or Alexion, bcTTM; Figure 1) has also been proposed as a drug candidate due to its improved stability compared to the ammonium derivative [13]. In 2023, BcTTM was removed from Astra-Zeneca’s clinical-phase pipeline after two unsuccessful phase-II trials at 15–60 mg per day in adults [14], a dose that was ineffective “to show that copper was actually eliminated from the body” [15]. A phase-III trial with bcTTM administered at 60 mg daily is, however, in progress [16]. Consequently, we decided to add bcTTM to the present comparative study, together with the clinically used drugs DPA and TETA, both in vitro and in the TX model of WD.

## 2. Materials and Methods

All solvents were purchased and used without purification. TDMQ20 was prepared as reported [17].

### 2.1. Aerobic Oxidation of Ascorbate

Aerobic oxidation of ascorbate in the presence of chelators was carried out in the experimental conditions reported [8].

### 2.2. Activity of Cu,Zn-SOD

The activity of Cu,Zn-SOD in the presence of copper chelators was monitored using a dedicated SOD kit supplied by Sigma-Aldrich (Saint-Quentin-Fallavier, France) as described [8], except that the mixture containing Cu,Zn-SOD and the chelator was pre-incubated for 1 h at 37 °C in a UV-vis cuvette before addition of the reagent working solution (WST-1) and enzyme working solution (xanthine oxidase). The production of formazan was monitored at 450 nm for 45 min at 37 °C under magnetic stirring.

### 2.3. Animals and Treatments

The animals used in the treatments included TX mice, provided by Sun Yat-Sen University (Guangzhou), and C57BL/6 mice, provided by the Guangdong experimental animal center (Guangzhou). The animals were in good health and had not been subjected to any previous procedure. After a 14-day acclimatization period, the 8-week-old TX mice (WD) were randomly distributed into 8 groups of 6 mice (3 males + 3 females, or 2 males + 4 females). The control group was made up of 3 male and 3 female C57BL/6 mice. Six mice per group is a necessary and sufficient sample size for a statistically significant analysis of the results. So, the number of animals used was limited to the strict minimum necessary to achieve the scientific validity of the results, in the framework of the 3Rs principle.

The mice in each group were then treated by intragastric gavage twice a day (every 12 h) for 14 consecutive days. For each treatment, the TX mice received TDMQ20 in NaCl 0.9 wt% at doses of either 0, 6.25, 12.5, or 25.0 mg/kg/ (corresponding to the daily doses 0, 12.5, 25.0, 50.0 mg/kg/d) for the WD, TDMQ-12.5, TDMQ-25, and TDMQ-50 groups, respectively. On the same schedule, the C57BL/6 mice received NaCl 0.9 wt%. The DPA groups were treated twice a day with DPA at 12.5 or 100 mg/kg in NaCl 0.9 wt% (daily dose = 25.0 or 200 mg/kg/d, respectively). The TETA groups were also treated twice a day with TETA at 12.5 or 100 mg/kg in NaCl 0.9 wt% (daily dose = 25 or 200 mg/kg/d, respectively). The TTM group was treated twice a day with bcTTM at 6.0 mg/kg in NaCl 0.9 wt% (daily dose = 12 mg/kg/d).

At the end of the treatment period, the mice (10-week-old) were placed in metabolic cages for 12 h in order to collect urine and feces. At the end of this 12 h period, the mice were anesthetized using 2.5% Avertin diluted in 0.9% NaCl at a dose of 125 μL/10 g body weight and sacrificed. Cardiac blood was collected and left to coagulate at room temperature for 30 min before centrifugation at 4 °C at 3000× *g* rpm for 10 min to obtain serum samples. During this 30 min period, liver, kidney, and serum were collected for copper dosage.

### 2.4. Cu Dosage in The Liver, Urine, and Kidneys

The copper concentrations in the urine, liver, and kidney were determined via inductively coupled plasma mass spectrometry (ICP-MS) on a ICAP-QC equipment (ThermoScientific, Waltham, MA, USA) [8]. All the samples of the six mice per group were used (n = 6), except in the following cases: (1) liver analysis: n = 5 in the group DPA-25 mg/kg (one sample lost), (2) urine analysis: n = 5 in groups WD, TDMQ20-25 mg/kg, DPA-25 mg/kg, and TTM-12 mg/kg; n = 4 in groups TDMQ20-50 mg/kg and DPA-200 mg/kg (failed to recover a sufficient volume of urine), and (3) kidney analysis: n = 4 in group TDMQ20-50 mg/kg; n = 5 in group DPA-25 mg/kg (identified experimental errors).

### 2.5. Apo- and Holo-CP in Mouse Serum

Apo- and holo-CP were quantified in mouse serum using non-denaturing Western blot conditions, as follows. The serum samples of 3 mice were analyzed, chosen at random from each group. Quantification of total proteins was carried out in each serum sample using a BCA kit (Beyotime, Haimen, China; Cat No. P0012S); each sample was then mixed at a 5/1 volume ratio of serum/non-denaturing and non-reducing loading buffer (Biosharp, Hefei, China; Cat No. BL529B), placed on ice for 30 min, and stored at −80 °C until processing for detection. The electrophoresis of proteins was then carried out on a commercially available 10% non-denaturing PAGE gel (Biosharp, Hefei; Cat No. BL1336A). A constant voltage of 60 V was applied for 30 min to bring the sample proteins to the starting position of the separating gel. Then, a constant voltage of 140 V was applied for 4 h to separate the proteins according to their molecular weight, shape, and electrical charge (the size marker was pre-staining Rainbow Protein Marker 10-180 KDa, ThermoFisher, Shanghai, China; Cat No. 26617). The above electrophoresis processes were carried out under ice bath conditions to prevent protein denaturation during electrophoresis. Under the condition of a constant current of 300 mA for 1 h, the separated proteins were transferred to a PolyVinyliDene Fluoride (PVDF) membrane, and the PVDF membrane was then blocked with 5% skimmed milk powder for 2 h. Then, the membranes were incubated overnight with the primary antibody at 4 °C (Ceruloplasmin Monoclonal antibodies, Proteintech (Wuhan Shi, China; Cat No. 66156-1-Ig, dilution 1/2500 and Albumin Monoclonal antibody, Proteintech, Cat No. 66051-1-Ig, dilution 1/5000). The protein membranes were washed 3 × 10 min with tris-buffered saline containing 0.1% Tween 20 (TBST). The secondary antibody was then incubated at room temperature for 1 h (Affinity brand Goat Anti-Mouse IgG (H+L) HRP- # S0002, dilution 1/10,000). The protein membranes were washed 3 × 10 min with TBST and then 1 × 5 min with tris-buffered saline (TBS). Finally, the protein membranes were developed with ECL Western blotting substrate (Solarbio, Beijing, China; Cat no. PE0010). The experiments were carried out in triplicate.

The equipment used for gel preparation and separation was a Mini-Double Vertical Electrophoresis System (Beijing Liuyi Instrument Factory, Beijing, China) with a DYY-7C Power Supply (Beijing Liuyi Instrument Factory). ECL development was carried out using a Peiqing JS-1070P chemiluminescence gel imaging analyzer (JS-1070P, Jiaozuo, China).

### 2.6. Ferroxidase Activity of CP in Serum

The ferroxidase activity of CP in the mouse serum was quantified for all 6 mice in each group using CP’s ability to catalyze 3,3′,5,5′-tetramethylbenzidine reduction to produce formazan, detected by its characteristic absorption at 645nm. A CP Activity Kit (Solarbio, Beijing, China; Cat No. BC1305) was used according to the supplier’s instructions.

### 2.7. Analysis of Liver ATP7B and CP

Analysis of liver ATP7B and CP in the mice was carried out using denaturing Western blot conditions, as follows. The serum samples of 3 mice were analyzed, chosen at random from each group. Quantification of total proteins was carried out for each serum sample using a BCA kit (Beyotime, Haimen, China; Cat No. P0012S), and each sample was then diluted to 3 mg/mL of proteins. The diluted samples were heated in a water bath at 95 °C for 10 min to denature proteins. The electrophoresis of proteins was then carried out on commercially available 8% sodium dodecyl sulfate (SDS) polyacrylamide gels (Biosharp, Hefei, China; Cat No. BL522A). A constant voltage of 60 V was applied for 30 min to bring the sample proteins to the starting position of the separator gel. Then, a constant voltage of 120 V was applied for 1 h to separate the proteins according to their molecular weights (the size marker was pre-staining Rainbow Protein Marker 10–180 KDa, ThermoFisher, Shanghai, China; Cat No. 26617). Under a constant current of 300 mA for 1 h, the separated proteins were transferred to a PolyVinyliDene Fluoride (PVDF) membrane, and the PVDF membrane was then blocked with 5% skimmed milk powder for 2 h. Then, the membranes were incubated overnight at 4 °C, with the primary antibody (Ceruloplasmin Polyclonal antibody, Proteintech, Wuhan Shi; Cat No. 21131-1-AP, dilution 1/1000, ATP7B-Specific Polyclonal antibody, Proteintech, Wuhan Shi; Cat No. 19786-1-AP, dilution 1/25,000, and beta Actin Antibody, Affinity, Cat No. AF7018, dilution 1/10,000). The membrane was washed 3 × 10 min with tris-buffered saline containing 0.1% Tween 20 (TBST). The secondary antibody was then incubated at room temperature for 1 h (Affinity brand Goat Anti-Rabbit IgG (H + L) HRP- # S0001, dilution 1/10,000). The membrane was washed 3 × 10 min with TBST, then for 1 × 5 min with tris-buffered saline (TBS). Finally, the membrane was developed with ECL Western blotting substrate (Solarbio, Beijing, China; Cat no. PE0010).

The equipment used for gel preparation and separation was a Mini-Sub Cell GT Horizontal Electrophoresis system (Bio-Rad, Hercules, CA, USA) with a DYY-7C Power Supply (Beijing Liuyi Instrument Factory). ECL development was carried out using a Peiqing JS-1070P chemiluminescence gel imaging analyzer (JS-1070P, Jiaozuo, China). The experiments were carried out in triplicate.

### 2.8. Hepatic CYP7A1 and CYP7B1 in Mice 

Hepatic CYP7A1 and CYP7B1 in the mice were analyzed by Tandem Mass Tag (TMT) LC-MS/MS. The livers of 3 mice were analyzed individually, chosen at random from each group. Liquid nitrogen was added to the frozen liver samples (20 mg), which were weighed in a 2 mL centrifuge tube. Sample lysing buffer, phosphatase inhibitor, and phenylmethylsulfonyl fluoride (PMSF) at a final concentration of 1 mM were then added. The liver samples were then ground twice in a cryostat at −35 °C for 120 s. The samples were then centrifuged at 12,000× *g* rpm for 10 min at 4 °C, and the supernatants were harvested. The quantification of total proteins was carried out using a BCA kit (ThermoScientific, Shanghai, China; Cat no. 23225). The volume corresponding to 50 μg of protein was taken from each liver sample and diluted to 0.5 mg/mL with cell lysis buffer (Beyotime, Haimen, China; Cat no. P0013G) before trypsinization. A TMTpro16 Labeling Kit (ThermoScientific, Shanghai; Cat no. A44520) was used to label the peptide fragments. LC-MS/MS was performed on these labeled liver samples using a Zorbax Extend-C18 column (2.1 × 150 mm, 5 μm, from Agilent, Santa Clara, CA, USA) at a flow rate of 300 μL/min. The eluent A was H_2_O containing 0.1 vol % of formic acid (FA), and the eluent B was acetonitrile/H_2_O/FA, 80/19.9/0.1, *v*/*v*/*v*). The elution gradient consisted of linear slope segments as follows: from A/B = 98/2 at t_0_ to A/B = 72/28 at 50 min, then A/B = 58/42 at 60 min, and then A/B = 10/90 at 65 min. The ratio A/B = 10/90 was then maintained until 75 min. The mass resolution of the first-level MS was set to 60,000, and the maximum injection time was 50 ms. Mass spectrometry scanning was set to the full scan charge-to-mass ratio *m*/*z* range of 350–1500. All MS/MS spectra were collected using high-energy collisional fragmentation in data-dependent positive ion mode. The resolution of MS/MS was set to 30,000, and the maximum ion injection time was 80 ms. The dynamic exclusion time was set to 30 s.

### 2.9. Data Analysis

Data analyses were carried out using SPSS Statistics software (version 26). Data are provided as mean values ± standard error of the mean (SEM). Statistical analyses were performed using a non-parametric test. Differences with *p* > 0.05 were considered not significant (ns), * *p* < 0.05, ** *p* < 0.01, and *** *p* < 0.001, n = 6 except otherwise stated. Graph presentations were drawn with Prism GraphPad 8.0.2.

## 3. Results

### 3.1. Copper Dosage in TX Mice After Treatment with Copper Chelators

**Figure 2:**
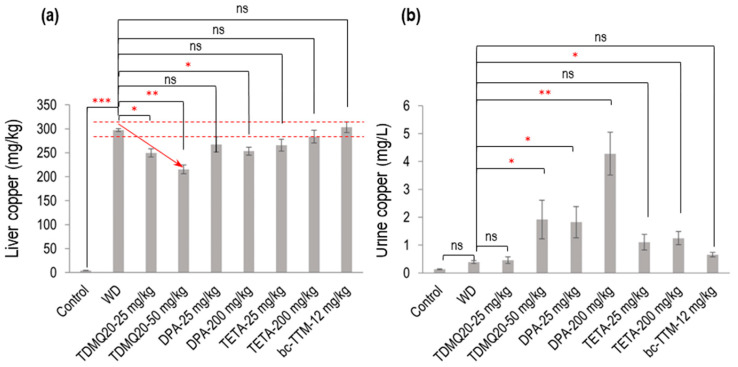
Dosage of copper in the liver (**a**) and urine (**b**) of TX mice (WD) after oral treatment for 14 days with TDMQ20 at 25 mg/kg/d or 50 mg/kg/d, compared with WD mice orally treated with DPA at 25 or 200 mg/kg/d, with TETA at 25 or 200 mg/kg/d, or with bcTTM at 12.0 mg/kg/d. Control mice are healthy C57BL/6 mice. The red arrow indicates the dose-dependent decrease in liver Cu concentration upon treatment with TDMQ20. Dashed red lines indicate the mean value obtained for WD mice ± 5%. The *p*-values with statistical significance for liver copper are as follows: control vs. WD < 0.001, WD vs. TDMQ20-25 mg/kg = 0.020, WD vs. TDMQ20-50 mg/kg = 0.001, and WD vs. DPA-200 mg/kg = 0.035. The *p*-values with statistical significance for urine copper are as follows: WD vs. TDMQ20-50 mg/kg = 0.030, WD vs. DPA-25 mg/kg = 0.016, WD vs. DPA-200 mg/kg = 0.001, and WD vs. TETA-200 mg/kg = 0.040. Groups TDMQ20-25 mg/kg and TDMQ20-50 mg/kg are similar to groups TDMQ20-M and TDMQ20-H, respectively, as reported [8]. Differences with *p* > 0.05 were considered not significant (ns), * *p* < 0.05, ** *p* < 0.01, and *** *p* < 0.001, n = 6 except otherwise stated in Table 1.

The eight-week-old TX mice (WD) were treated per os for 14 days using either TDMQ20 at daily doses of 25.0 and 50.0 mg/kg (in NaCl 0.9%); DPA at daily doses of 25 mg/kg for direct comparison with TDMQ20; or 200 mg/kg/d, which is the reference dose of DPA in TX mice [18]. In all cases, half the daily doses were administered in the mornings and evenings. For comparison, the TX mice were also treated either with TETA at the same doses as DPA (i.e., 25 or 200 mg/kg/d) or with bcTTM at 12 mg/kg/d [18]. Urine samples were collected over a 12 h period following the final administration. The mice were then sacrificed at d15, and copper concentrations in the liver were determined via ICP-MS and compared to those of the normal (healthy) C57BL/6 mice used as controls. The mean values of each animal group are reported in Table 1 and Figure 2. Individual mouse dosages are reported in the Supporting Information (Table S1).

As expected, the level of copper in the livers of the untreated WD mice (Table 1, WD group) was dramatically higher than that in the healthy mice (control group), with concentrations at 4.3 mg/kg and 298 mg/kg, respectively. Treatment with TDMQ20 at a daily dose of 25 or 50 mg/kg for 14 days induced a significant dose-dependent decrease in hepatic copper loading (−16% at 25 mg/kg, −28% at 50 mg/kg). DPA administered at 200 mg/kg exhibited an effect equivalent to TDMQ20 at 25 mg/kg (−15%). This result is consistent with our previous report, confirming that a dose of DPA 8 times higher than that of TDMQ20 is required to produce a comparable decrease in hepatic Cu [8]. Moreover, DPA at 25 mg/kg induced only a 10% reduction in hepatic copper loading, which was not statistically significant. The administration of TETA at 25 or 200 mg/kg/d induced a reduction in hepatic copper concentration ranging from 5% to 11% compared with the untreated mice (Table 1). This decrease was not statistically significant and not dose-dependent (Figure 2a). BcTTM at the daily dose of 12 mg/kg failed to decrease the liver Cu concentration in the TX mice compared with the untreated animals (Table 1, Figure 2a). Treatment with TDMQ20 induced a significant increase in the urine excretion of Cu with respect to the WD group, especially at 50 mg/kg (× 4.9 versus WD, Table 1, Figure 2b). This effect was similar to that of DPA at 25 mg/kg (× 4.7 vs. WD) and lower than that of DPA at 200 mg/kg (× 11 vs. WD). TETA at the high dose of 200 mg/kg exhibited a lower effect than that of TDMQ20 at 50 mg/kg/d (a dose 4 times lower) or DPA at 25 mg/kg/d (8 times lower). BcTTM 12 mg/kg did not significantly improve the urinary excretion of Cu (× 1.7 versus WD). Consequently, DPA at 200 mg/kg/d is the most efficient treatment in improving the urinary excretion of Cu. Conversely, it was reported that TDMQ20 significantly increased fecal elimination of copper in a dose-dependent manner in untreated WD mice, which was not the case in WD mice treated with DPA at 200 mg/kg [8], suggesting different copper excretion routes for these two drugs. It is worth noting that in humans, it was reported that bcTTM strongly inhibited the dietary uptake of ^64^Cu in the intestinal lumen, resulting in increased fecal excretion, but did not increase biliary excretion of ^64^Cu in healthy volunteers [19].

**Figure 3:**
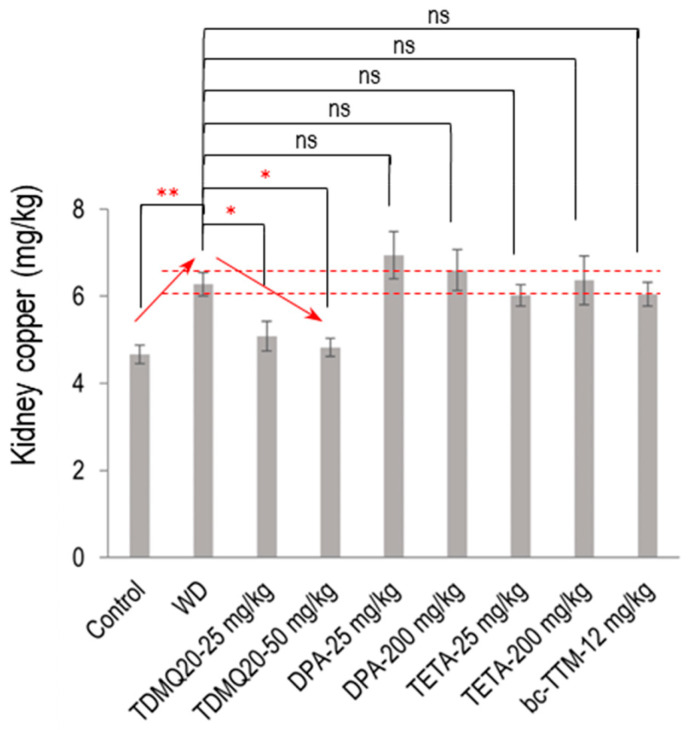
Dosage of copper in the kidneys of TX mice (WD) after oral treatment for 14 days with TDMQ20 at 25 mg/kg/d or 50 mg/kg/d, compared with WD mice orally treated with DPA at 25 or 200 mg/kg/d, with TETA at 25 or 200 mg/kg/d, or with bcTTM at 12.0 mg/kg/d. Control mice are healthy C57BL/6 mice. Red arrows indicate the dose-dependent decrease in kidney Cu concentration upon treatment with TDMQ20. Dashed red lines indicate the mean value obtained for WD mice ± 5%. The *p*-values with statistical significance for kidney copper are as follows: control vs. WD = 0.007, WD vs. TDMQ20-25 mg/kg = 0.047, and WD vs. TDMQ20-50 mg/kg = 0.031. Groups TDMQ20-25 mg/kg and TDMQ20-50 mg/kg are similar to groups TDMQ20-M and TDMQ20-H, respectively, as reported in [8]. Differences with *p* > 0.05 were considered not significant (ns), * *p* < 0.05, and ** *p* < 0.01, n = 6, except for the TDMQ20-50 mg/kg group (n = 4) and for the DPA-25 mg/kg group (n = 5).

The mean concentration of Cu in the kidneys of the WD mice was significantly increased compared to the control mice (6.28 mg/kg compared to 4.66 mg/kg, an increase of +35%, Figure 3). Individual mouse dosages are reported in the Supporting Information, Table S2. This copper concentration was normalized (4.83 mg/kg) after treatment with TDMQ20 at the daily dose of 50 mg/kg. Conversely, the copper level was not significantly decreased by any other treatment, namely, DPA, TETA, or bcTTM, remaining in the range of 6–7 mg/kg. This result suggests a possible storage/accumulation of Cu in the kidneys of WD animals, a situation that was reversed by treatment with TDMQ20, but not with the other drugs.

### 3.2. Serum Ceruloplasmin in TX Mice

#### 3.2.1. Quantity of Apo-CP and Holo-CP in the Serum

**Figure 4:**
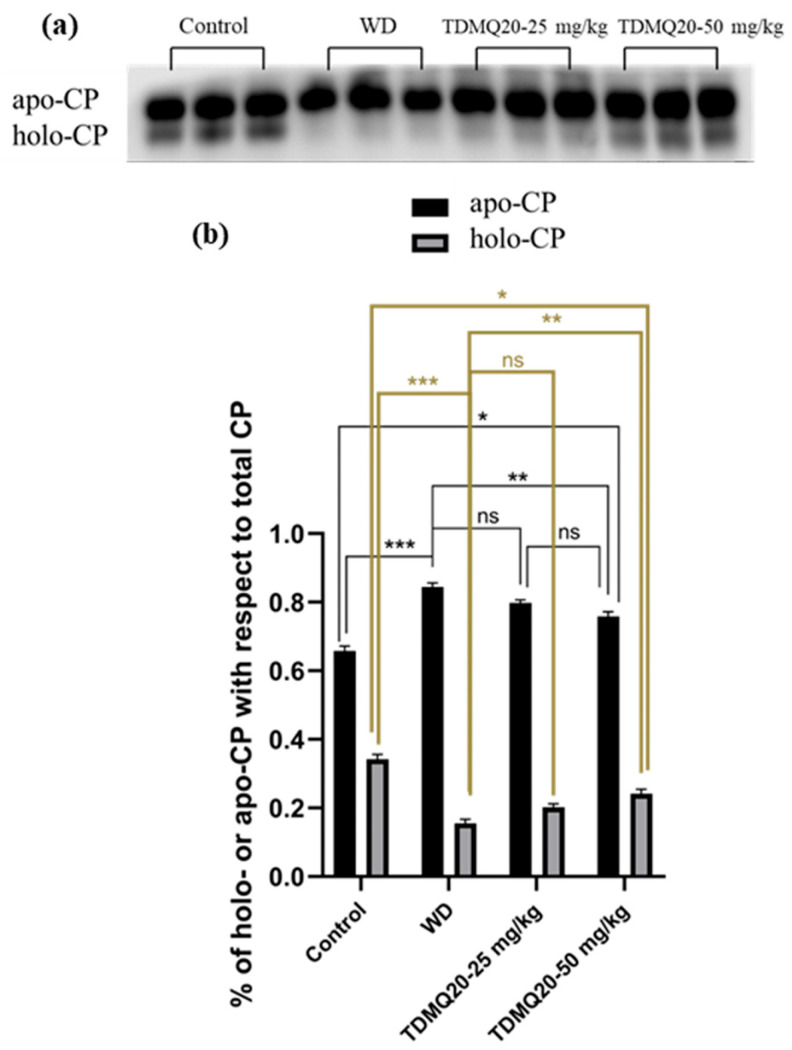
(**a**) Representative example of a non-denatured Western blot analysis of serum ceruloplasmin (CP, 30 µg of total proteins deposited in each lane). (**b**): Proportions of apo-CP (black bars) and holo-CP (grey bars) with respect to total CP; the results depicted in panel (**b**) are the mean value ± SEM of three protein deposits. Groups TDMQ20-25 mg/kg and TDMQ20-50 mg/kg are similar to groups TDMQ20-M and TDMQ20-H in [8]. Not significant (ns) when *p* > 0.05. The *p*-values with statistical significance for holo-CP (**c**) are as follows: control vs. WD < 0.001, WD vs. TDMQ20-25 mg/kg = 0.002, WD vs. TDMQ20-50 mg/kg < 0.001, and control vs. TDMQ20-50 mg/kg = 0.001. Individual data reported in Table S3. The experiment was carried out three times with comparable results. Differences with *p* > 0.05 were considered not significant (ns), * *p* < 0.05, ** *p* < 0.01, and *** *p* < 0.001.

The concentration of ceruloplasmin in the serum of the WD mice was reported to be significantly lower than that of the control mice. This impairment was fully reversed by oral treatment with TDMQ20 at 25 or 50 mg/kg/d [8]. It has been reported, however, that (i) apo-CP is the major component of total CP in mammalian blood plasma [20], despite the fact that its turnover (removal from the circulation) is more rapid than that of the holo form [21], and (ii) the holo-CP concentrations in mouse serum increase in parallel with the copper concentration [22]. We thus investigated the proportions of holo-ceruloplasmin (holo-CP) and apo-ceruloplasmin (apo-CP) by analysis of mouse serum proteins by PAGE electrophoresis in non-denaturing conditions. The proportion of apo-CP and holo-CP with respect to the total amount of CP was calculated for each gel. The results reported in Figure 4 indicate that holo-CP in the WD serum was only 16% of CP, compared to 34% in the controls. The treatment of the WD mice with TDMQ20 resulted in significant and dose-dependent increases in the proportion of holo-CP (+25% at 25 mg/kg/d and 50% at 50 mg/kg/d, Figure 4b, grey bars).

In addition, the amount of apo-CP in the mouse serum, measured as the apo-CP/albumin ratio, was not significantly impaired in the WD mice compared to the controls, and it did not change upon treatment with TDMQ20 up to 50 mg/kg/d (Table S4, Figure S1). Conversely, holo-CP was drastically lower in the WD mice compared to the controls. It is worth noting that the treatment with TDMQ20 resulted in a significant and dose-dependent increase in the holo-CP/albumin ratio. These results indicate a significant mobilization of Cu after oral treatment with TDMQ20 at 50 mg/kg/d.

#### 3.2.2. Ferroxidase Activity of CP in Serum

**Figure 5:**
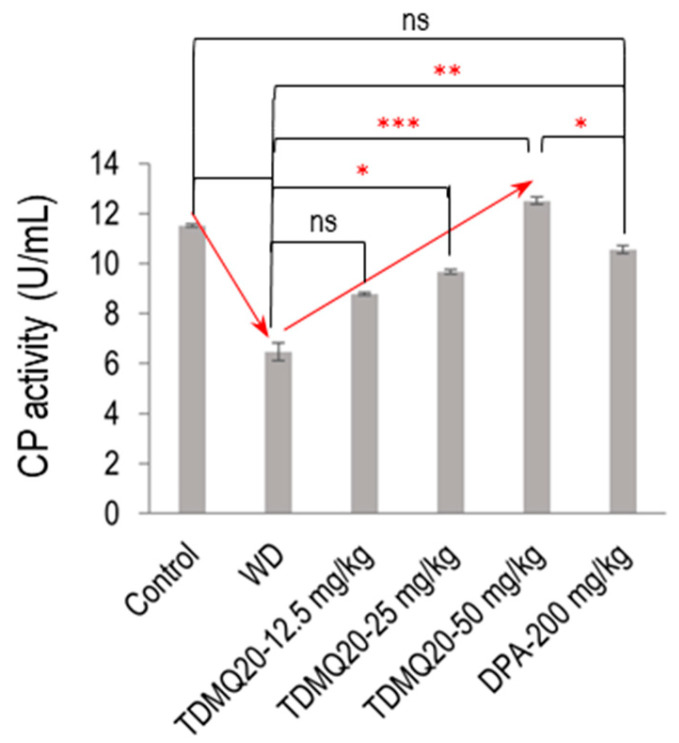
Serum ferroxidase activity in each group of mice. The result is the mean value ± SEM of six mice in each group. Differences with *p* > 0.05 were considered as not significant (ns), * *p* < 0.05, ** *p* < 0.01, and *** *p* < 0.001. The *p*-values with statistical significance are as follows: control vs. WD < 0.001, WD vs. TDMQ20-25 mg/kg = 0.049, WD vs. TDMQ20-50 mg/kg < 0.001, TDMQ20-50 mg/kg < 0.001, WD vs. DPA-200 mg/kg = 0.003, and TDMQ20-50 mg/kg vs. DPA-200 mg/kg = 0.049. Groups TDMQ20-12.5 mg/kg, TDMQ20-25 mg/kg, and TDMQ20-50 mg/kg are similar to groups TDMQ20-L, TDMQ20-M, and TDMQ20-H, respectively, as reported in [8].

In the mammalian plasma, 95% of the copper ions are associated with CP. As the principal plasma ferroxidase, CP is responsible for the production of Fe(III), the form of iron that can be bound by transferrin and ferritin [22]. So, to confirm the functional status of serum holo-CP upon the treatment of WD mice with TDMQ20, the ferroxidase activity of CP was evaluated using a *p*-phenylenediamine substrate, namely, 3,3′,5,5′-tetramethylbenzidine [23]. The results are depicted in Figure 5 (raw data for six independent dosages are reported in Table S5). The oxidase activity of the WD mouse serum was 6.47 ± 0.36 U/mL, compared to 11.51 ± 0.07 U/mL in the control serum (−44%). It should be noted that this result is consistent with the lower proportion of holo-CP, which was roughly half in the WD mice compared to the controls (Figure 4b). The treatment of the WD mice with TDMQ20 resulted in a dose-dependent increase in the enzymatic activity of CP, which was nearly restored at the daily dose of 25 mg/kg (84% of the control value) and fully restored at the daily dose of 50 mg/kg. Conversely, the treatment with DPA at the much higher dose of 200 mg/kg/d was necessary to produce a similar effect (Figure 5).

Due to its ferroxidase activity, ceruloplasmin is a physiologically relevant inhibitor of myeloperoxidase, suggesting that it should provide a protective shield against inadvertent oxidative stress induced by myeloperoxidase during inflammation [24,25]. In conditions of oxidative stress, restored CP activity may alleviate liver inflammation (hepatitis) and subsequent fibrosis, as well as neuroinflammation in WD patients [26,27]. It is worth noting that in the cortex of two different mouse models of Alzheimer’s disease, TDMQ20 was found to reduce the level of malondialdehyde (MDA), a marker of the occurrence of lipid damage induced by peroxides [7]. This result is consistent with the restoration of ferroxidase activity of CP by TDMQ20 and the subsequent lower activity of myeloperoxidase. For related reasons, it was proposed that CP may play a protective role against neurodegenerative diseases [28,29,30].

### 3.3. Liver Proteins in TX Mice

#### 3.3.1. ATP7B and CP

**Figure 6:**
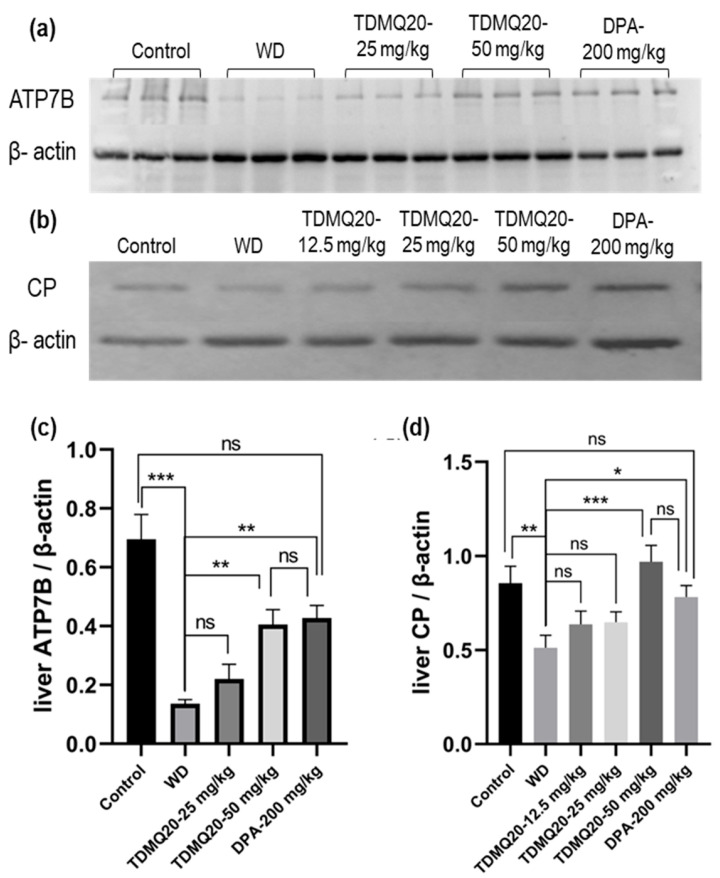
Representative example of a denatured Western blot analysis of liver *ATP7B* (**a**) and CP (**b**). The quantification of liver *ATP7B* and CP was carried out using β-actin as an internal standard (panels (**c**) and (**d**), respectively). The results are the mean value ± SEM of 3 mice in each group. Differences with *p* > 0.05 were considered as not significant (ns), * *p* < 0.05, ** *p* < 0.01, and *** *p* < 0.001. The *p*-values with statistical significance are as follows: for ATP7B/β-actin: control vs. WD < 0.001, 0.07, WD vs. TDMQ20-50 mg/kg = 0.003, and WD vs. DPA-200 mg/kg = 0.001; for CP/β-actin: control vs. WD = 0.007, WD vs. TDMQ20-50 mg/kg < 0.001, and WD vs. DPA-200 mg/kg = 0.017. The experiment was carried out three times with similar results.

As major proteins involved in WD, the levels of ATP7B and CP in the livers of the WD mice after treatment with TDMQ20 or DPA were analyzed by Western blot analysis after denaturing SDS-PAGE. The results are reported in Figure 6 and Table S6. As expected, the hepatic level of the ATP7B protein in the WD mice was drastically lower than that of the control mice. This concentration was significantly higher in the WD mice treated with TDMQ20 at 50 mg/kg/d, very similar to that of the WD mice treated with DPA at 200 mg/kg/d, and close to that of the control mice (Figure 6a,c).

The level of total CP (apo- + holo-protein) in the livers of the control mice was also significantly higher than that of the WD group. The treatment with TDMQ20 at 12.5 mg/kg/d and 25 mg/kg/d resulted in an increasing trend, but the results were not statistically significant. However, at the dose of 50 mg/kg/d, the hepatic concentration of CP returned to the value of the control group and was slightly higher than that of the mice treated with DPA (Figure 6b,d). These results underline the fact that the administration of TDMQ20 promotes the synthesis of ATP7B and CP in the livers of TX mice at a dose four times lower than that of DPA. This feature is expected to facilitate the excretion of copper by the liver.

#### 3.3.2. Other Proteins in the Liver, Mamely, CYP7A1 and CYP7B1, and the Relation with Lipid Metabolism

In the *Atp7b*^−/−^ mouse model of WD, copper down-regulates lipid metabolism, particularly cholesterol biosynthesis [31], and patients with hepatic symptoms exhibit lower total cholesterol serum concentration than others [32]. Steatosis is also frequent in patients with WD, and there is a trend for higher copper levels in patients with steatohepatitis; however, the direct link between copper increase and steatosis in WD remains unclear [33,34].

**Figure 7:**
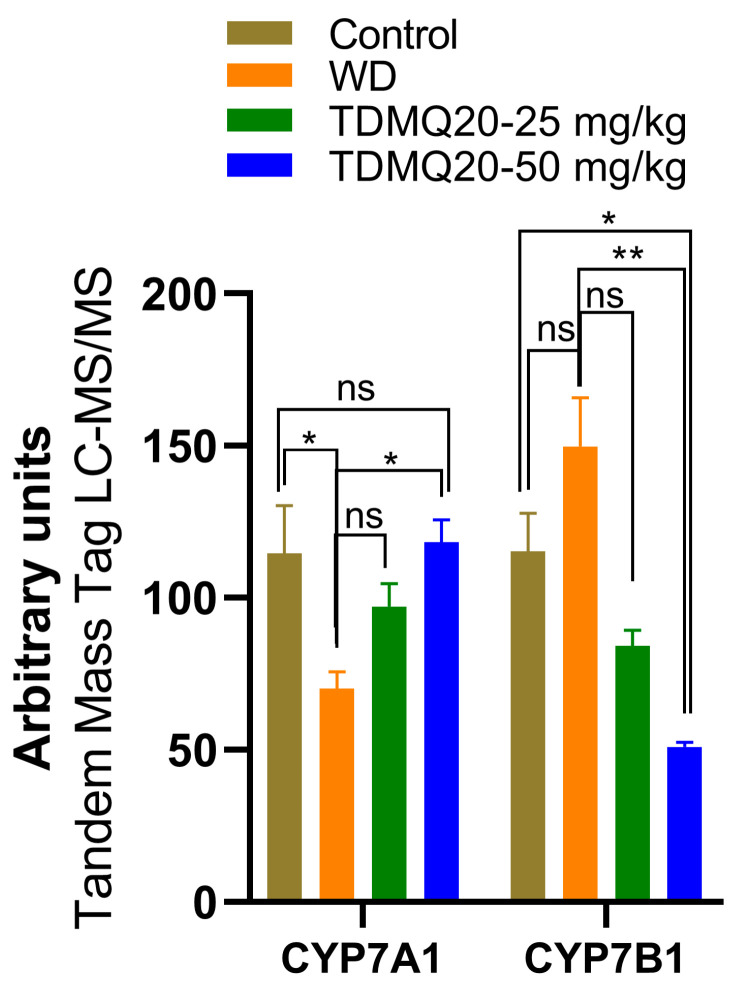
Liver proteomics analysis of CYP7A1 and CYP7B1 enzymes involved in bile acid and salt synthesis. The *p*-values with statistical significance are as follows: for CYP7A1: control vs. WD = 0.017 and WD vs. TDMQ20-50 mg/kg = 0.013; for CYP7B1: control vs. TDMQ20-50 mg/kg = 0.031 and WD vs. TDMQ20-50 mg/kg = 0.003. Differences with *p* > 0.05 were considered not significant (ns), * *p* < 0.05, and ** *p* < 0.01.

In a preliminary proteomics study of the livers of the TX mice, we found that the level of CYP7A1 is drastically lower in the TX mice compared to the controls, but that TDMQ20 at 50 mg/kg/d can up-regulate it (Figure 7 and Table S7). The cytochrome P450 isozyme CYP7A1 catalyzes the hydroxylation of cholesterol to 7α-hydroxycholesterol, playing a critical role in the homeostasis of mammalian cholesterol. This reaction is also the first and rate-limiting step in bile acid synthesis [35,36].

Consequently, the recovery of normal CYP7A1 levels in the TX mice under TDMQ20 treatment is consistent with both the reduction in steatosis in the liver and the activation of bile excretion of Cu that we recently reported in TX mice treated with TDMQ20 [8].

Contrary to CYP7A1, CYP7B1 (also known as 25-hydroxycholesterol 7α-hydroxylase or oxysterol 7α-hydroxylase) is up-regulated in the livers of the TX mice compared to the controls (Figure 7). The increase in CYP7B1 in the livers of the WD mice is reversed after treatment with TDMQ20 (Figure 7). This enzyme likely plays a minor role in total bile acid synthesis, but it is mainly involved in the 7α-hydroxylation of other steroid substrates, such as 25-hydroxycholesterol and the neuro-steroids dehydroepiandrosterone and pregnenolone [37,38,39]. To the best of our knowledge, the involvement of neuro-steroids in the neurological presentation of WD has only been poorly investigated up to now [40].

### 3.4. Inhibition of Cu,Zn-SOD by Copper Chelators In Vitro

**Figure 8:**
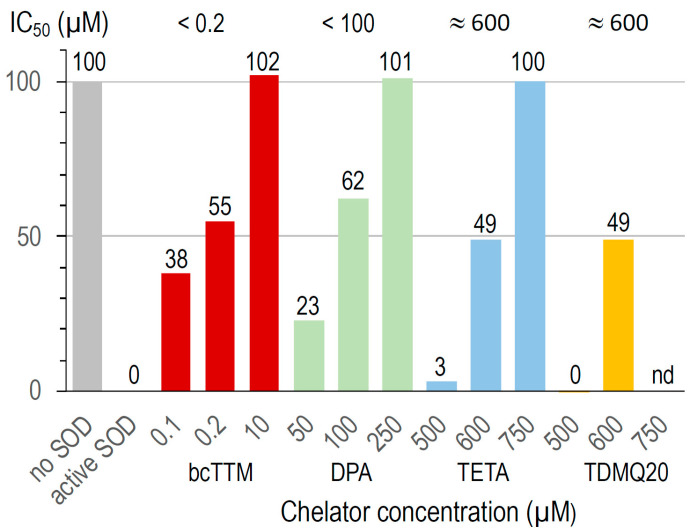
Inactivation of Cu,Zn-SOD in the presence of several concentrations of bcTTM 0.1–10 µM (red), DPA 50–250 µM (green), TETA 500–750 µM (blue), and TDMQ20 500–600 µM (yellow). TDMQ20 was not tested at concentrations higher than 600 µM due to its lack of solubility in the experimental medium of the Cu,Zn-SOD kit.

The activity of bovine Cu,Zn-SOD was measured in the presence of Cu chelators using a dedicated SOD kit. The superoxide anion produced by the xanthine/xanthine oxidase system reduces a tetrazolium salt to formazan, whose production is monitored by UV–visible spectroscopy (450 nm). A high formazan concentration reflects a high superoxide concentration and, consequently, high Cu,Zn-SOD inhibition. This assay was carried out at increasing concentrations of TDMQ20 and compared to the other chelators bcTTM, DPA, and TETA. The results are summarized in Figure 8.

In the conditions used, bcTTM inhibited Cu,Zn-SOD at a very low concentration, with an IC_50_ value below 0.2 µM (red bars). In fact, the *K*_aff_ value of bcTTM for Cu (4 × 10^19^ M^−1^ [41]) has been estimated to be much higher than that of SOD (10^17^ M^−1^ [42]), leading to the de-coppering of SOD and subsequent loss of enzymatic activity. Accordingly, TTM was reported as an antitumor copper-binding drug that attenuates angiogenesis and tumor cell proliferation through the inhibition of superoxide dismutase [43,44].

The IC_50_ value of DPA for the inactivation of Cu,Zn-SOD is in the range 50–100 µM (green bars). This value may explain the fact that the erythrocyte Cu,Zn-SOD is significantly decreased in patients undergoing long-term treatment with penicillamine [45]. The IC_50_ values of TETA and TDMQ20 were similar, close to 600 µM (blue and yellow bars, respectively). In fact, contrary to bcTTM and DPA, these drugs did not significantly inhibit SOD below 500 µM. However, one should note that the active concentration of TETA in the mice was 200 mg/kg, while that of TDMQ20 required to produce a comparable effect was 25–50 mg/kg, i.e., 4 or 8 times lower. So, TDMQ20 has a significant advantage over TETA in this respect since much lower doses should be used in vivo.

### 3.5. Aerobic Oxidation of Ascorbate by Copper Chelators In Vitro

**Figure 9:**
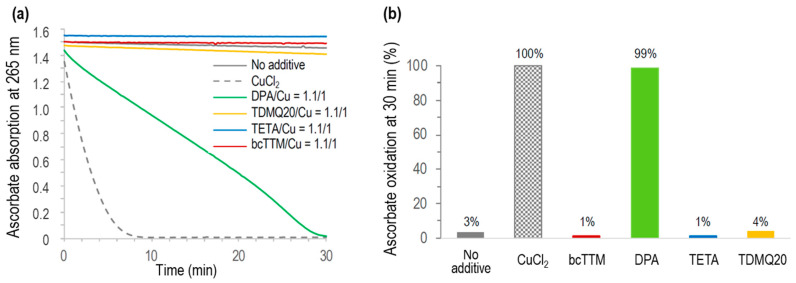
(**a**) Kinetics of ascorbate oxidation in the presence of CuCl_2_ alone (dashed grey), or CuCl_2_ associated with bcTTM (red), DPA (green), TETA (blue), or TDMQ20 (yellow), compared with ascorbate autoxidation (grey full trace). Drug/CuCl_2_ molar ratio = 1.1/1. (**b**) Percentage of ascorbate oxidation at 30 min.

The Cu-induced ascorbate oxidation in the presence of copper chelators (chelator/Cu molar ratio = 1.1/1) was monitored by UV–visible spectroscopy at 265 nm, as a classical test to detect in vitro ROS production induced by Cu(II) in the presence of molecular oxygen [46]. The results are reported in Figure 9. As expected, ascorbate was fully oxidized in the presence of CuCl_2_ after a few minutes (<10 min, Figure 9a, grey dashed trace) while autoxidation in the absence of Cu was only 3% after 30 min (no additive, Figure 9b). In the presence of DPA associated with Cu^2+^, the full oxidation of ascorbate was achieved in 30 min, consistent with already reported data [8]. The oxidation of ascorbate in the presence of any other used chelator, TETA, TDMQ20, or bcTTM, was negligible in these conditions (below 4% after 30 min, similar to autoxidation). So, like TDMQ20, TETA and bcTTM fully inhibited ascorbate oxidation in the presence of copper ions at a ligand/metal (L/M) molar ratio = 1/1 by sequestering Cu in a redox-inactive form.

Conversely, DPA did not inhibit this model of oxidative stress, even in excess of the chelator (DPA/Cu = 2/1) [8], suggesting that the copper complexes of DPA should induce oxidative stress in vivo, while other chelators should not. In fact, it was reported that DPA inactivates catalase through the reducing activity of its thiol functionality, thus disturbing H_2_O_2_ homeostasis. This process may explain some of the deleterious effects observed with DPA [47].

For bcTTM, a bcTTM/Cu molar ratio of 0.5/1 was also inefficient in oxidizing ascorbate (Figure S2), suggesting the formation of a redox-inactive complex of bcTTM containing at least two copper ions per ligand.

## 4. General Discussion and Conclusions

The commonly used drugs DPA or TETA both cause a very high incidence (10–50%) of neurological worsening in WD patients. This feature has been correlated with Cu-induced oxidative stress, which was markedly increased in the striatum of TX mice during DPA administration [18]. Conversely, complexation and retention of Cu by bcTTM in the bloodstream have been reported to protect against cerebral uptake of bioavailable copper, which may explain why bcTTM appears less likely to cause neurological worsening [19], despite its intrinsic toxicity (see below). These three chelators, either used in the clinic or in clinical development, have been compared with TDMQ20, a specific copper chelator able to improve the memory status of mouse models of Alzheimer’s disease [7]. TDMQ20 has been found safe in long-term administration in mice (45 doses of 10 mg/kg over a period of 3 months) [7].

### 4.1. Structures of the Copper Complexes of DPA, TETA, TTM, and TDMQ20

To produce suitable drug candidates capable of specifically mediating the excretion of copper or restoring its homeostasis, it is essential to take into consideration the basic coordination chemistry of this metal, for example, the selectivity of these ligands for Cu with respect to several other metals and the coordination sphere of the metal in the resulting Cu(II) complexes.

DPA and TETA have been reported to coordinate, with high affinity, a wide variety of metal ions (including biologically relevant ones, such as Cu, Zn, Fe, Ni, and Co) [48,49]. The metal complexes of DPA can exhibit discrete structures with stoichiometry L/M = 2/1 [50,51], as well as polymetallic cluster structures [52,53]. At physiological pH, DPA forms a copper complex with a mixed valence cluster containing Cu(I), Cu(II), and Cu^I^-S-Cu^II^ bridges [54]. In addition, the existence of ternary complexes involving other amino acids, such as histidine or methionine [55,56], or proteins [57] may explain the inhibition of metalloenzymes by DPA [45].

Contrary to DPA, TETA forms an octahedral complex with copper, with L/M stoichiometry = 1/1, where the four nitrogen atoms of the ligand chelate the Cu^II^ ion in a square planar geometry, the two apical positions being occupied by counter anions [58]. However, this ligand can also give rise to a hetero-binuclear Cu,Zn complex, where both metal ions are penta-coordinated and connected through an imidazolato bridge [59]. The versatile structures of the metal complexes of DPA and TETA in vivo may limit their efficiency and selectivity for the excretion of copper, thus accounting for the toxic side effects of these chelators.

Unlike DPA and TETA, TTM has been reported to have no significant affinity for Fe, Mn, Zn, Ca, or Mg [43]. However, while ammonium and potassium tetrathiomolybdates have been characterized as monomers by X-ray powder diffraction, the so-called tetrathiomolybdates of cobalt(II), nickel (II), copper (II), and zinc are in fact mixtures of the sulfides of these metals with molybdenum trisulfide, containing no thiomolybdate ion [60]. Conversely, the reaction of tetraethylammonium thiomolybdate with Cu^I^Cl provided a cluster based on a *bis*-cubane with [MoCu_2_S_4_] and [MoCu_3_S_4_] structures [61]. A cluster consisting of a tetrahedral MoS_4_ core enveloped by an octahedral array of six copper atoms, forming an MoS_4_Cu_6_ aggregate, has also been reported [62]. So, the tendency of TTM to behave as a bridge between metal centers and to form clusters or oligomeric structures may hamper its ability to efficiently excrete Cu in WD. Metallic aggregates can precipitate in physiological conditions and are more difficult to eliminate from organs than well-defined discrete metal–ligand complexes [63].

TDMQ20 chelates Cu(II) with high affinity (log *K*_app_ = 16.5 at pH 7.4), in a discrete complex with stoichiometry L/M = 1/1 [17]. This is clearly due to the N4-tetradentate structure of the ligand, offering a nearly perfect square planar coordination of the metal center, which is the preferred structure of Cu(II) complexes [64,65]. Consequently, TDMQ20 has a much lower affinity for Zn(II) (log *K*_app_ = 4.2 at pH 7.4), whose competitive coordination should be avoided in vivo since zinc ions are essential in zinc finger proteins as well as many allosteric proteins. Moreover, TDMQ20 has no significant affinity for Cu(I) and is consequently unable to induce redox stress by the Fenton-like reductive activation of dioxygen [17]. In addition, this chelator has no significant affinity for Fe(III), Ca(II), Mg(II) [65], or Fe(II) [66]. Owing to these properties, TDMQ20 does not generate TDMQ20–Cu–X ternary complexes [64,65] and does not inhibit Cu,Zn-SOD, tyrosinase, or other metal-containing cofactors in vitro [67].

### 4.2. Molybdenum Toxicity

Since molybdenum was found to be toxic in ruminants [10], the human safety of TTM is obviously a matter of question. In fact, several toxic effects have been reported in WD patients treated with TTM and in animals, including bone marrow depression [68], elevated liver enzymes [11], skeletal abnormalities [69], vomiting [70], and critical effects on reproduction and fetal development [71]. So, the tolerable daily intake (TDI) of molybdenum for adult human chronic exposure was estimated at 0.009 mg of Mo/kg. Based on the daily dose of 12 mg/kg of bcTTM tested in mouse models, the predictable efficient dose in humans is expected to be roughly 1 mg/kg [72], corresponding to a daily dose of 0.22 mg/kg of Mo, i.e., 25 times higher than the calculated TDI. In fact, the daily dose of ammonium TTM evaluated in adult patients with WD was in the range of 120-240 mg [73,74], corresponding to 0.6–1.2 mg Mo/kg (body weight = 70 kg), i.e., 70–140 times higher than the TDI. Moreover, the accumulation of molybdenum in major organs (liver, spleen, kidneys, adrenals, brain) was evidenced after repeated oral administration of bcTTM to Sprague Dawley rats (15 or 60 mg/kg/d for 12 weeks) [75]. This result is consistent with a previous report where Long-Evans Cinnamon (LEC) rats (which carry a mutation of the *ATP7B* gene leading to Cu overload) were treated by intraperitoneal injection of tetrathiomolybdate. Microscopic examination of the livers of the animals indicated that both copper and molybdenum colocalized in lysosomes as diverse copper-molybdenum-sulfur clusters [76]. Consistently, molybdenum clearance was much lower in the LEC rats than in the control strain (Long-Evans Agouti [LEA] rats), which had normal copper metabolism [77]. This suggests a probable higher toxicity of TTM in patients with WD than in healthy persons. In fact, treatment of the *Atp*7b^−/−^ mouse model (10 mg/kg/d, 4 weeks) with bcTTM compromises mitochondrial integrity [78].

Besides the inhibition of Cu,Zn-SOD, TTM also inhibits several copper enzymes or proteins [79], especially proteins that regulate copper trafficking, thus preventing metal transfer functions. For example, the high stability of the TTM complex with the metallochaperone Atx1 arises from the formation of a sulfur-bridged copper-molybdenum cluster [S_6_Cu_4_MoS_4_] reminiscent of those found in molybdenum and iron sulfur proteins [80]. TTM also forms a ternary complex with Cu and albumin, retaining Cu in the bloodstream [19].

All these results suggest that the mechanism of action of TTM may be a decrease in the bioavailability of Cu (or a change in its redox properties) rather than an increase in its excretion [15]. For a disease where lifelong treatment is indicated, the progressive and deleterious accumulation of Mo may have serious clinical consequences.

### 4.3. Biological and Biochemical Comparison of DPA, TETA, TTM, and TDMQ20

Based on our results and relevant bibliographic data, a comparison of DPA, TETA, TTM, and TDMQ20 as copper chelators to treat WD is summarized in Table 2. First, when used at a daily dosage as low as 25 mg/kg, TDMQ20 decreased the copper loading in the livers and the kidneys of the TX mice (Table 2, entries 6 and 8, respectively). Only the clinically used DPA can decrease Cu in the liver, and only when used at 8 times the dose (i.e., 200 mg/kg). When used at 200 mg/kg/d, DPA (and, to a lesser extent, TETA) increased the urinary excretion of Cu, while TTM failed to increase Cu concentrations in urine. However, the treatment with DPA did not improve the fecal excretion of Cu (entry 9). TTM (12 mg/kg) promoted the fecal excretion of Cu, but it has been demonstrated that this effect is due to the inhibition of the dietary uptake of Cu in the intestinal lumen, rather than increased biliary excretion [19]. Conversely, the increase in both the urinary and fecal excretion of Cu induced by TDMQ20 showed a significant and dose-dependent effect in the dose range of 12–25 mg/kg. This indicates the ability of TDMQ20 to rescue the fecal excretion of Cu, which is the normal route in healthy individuals.

TDMQ20 increased holo-CP and ferroxidase activity of CP in the serum of the treated TX mice (entries 11 and 12, respectively), while the CP and ATP7B levels were also increased in the livers (entries 13 and 14, respectively), consistent with the exportation of hepatic Cu by the chelator. In addition, the normalization of the concentrations of liver CYP7A and CYP7B after TDMQ20 treatment at an effective concentration (entry 15) is consistent with the decrease in liver steatosis (entry 10) and may reveal a possible target for the therapy of WD, which has not been investigated up to now.

Compared with the other ligands, TDMQ20 is the only chelator that efficiently mediates the excretion of Cu and the restoration of active CP levels at low doses. Such efficacy is related to the design of this chelator, which specifically coordinates Cu(II) as a discrete and soluble complex, without the participation of any other metal or ligand (entries 1 and 2). Conversely, DPA, TETA, and TTM give rise to various complexes with Cu, often with oligomeric or cluster structures, which can be retained in blood circulation or sequestered by proteins.

To conclude, taking into consideration all the advantages of TDMQ20 compared to the other ligands and its lack of toxicity during long-term administration in mice [7], the drug candidate TDMQ20 appears to be a first-class challenger with respect to DPA, TETA, and bcTTM.

## Figures and Tables

**Table 1:** Concentration of copper in control mice, TX mice (WD), and TX mice treated with TDMQ20 or DPA or TETA or bcTTM, as mean values for 6 mice of each group ± SEM, calculated using SPSS Statistics software, v. 26. ^a^ Decrease in Cu concentration in treated mice with respect to WD, calculated as Δ = [1 − (Cu in treated group/Cu in WD group)] × 100. ^b^ Increase in Cu concentration in treated mice with respect to WD, calculated as (Cu in treated group/Cu in WD group). ^c^ n = 5. ^d^ n = 4.

Group n = 6	Mean Cu in Liver ± SEM (mg/kg)	Δ Versus WD ^a^	Mean Cu in Urine ± SEM (mg/L)	Δ Versus WD ^b^
Control	4.3 ± 0.3		0.13 ± 0.05	
WD (untreated)	298 ± 3.1	-	0.39 ± 0.12 ^c^	-
25 mg/kg TDMQ20	250 ± 8.7	−16%	0.46 ± 0.27 ^c^	× 1.2
50 mg/kg TDMQ20	215 ± 0.4	−28%	1.92 ± 1.38 ^d^	×4.9
25 mg/kg DPA	267 ± 15.6 ^c^	−10%	1.82 ± 1.25 ^c^	×4.7
200 mg/kg DPA	254 ± 8.3	−15%	4.28 ± 1.53 ^d^	×11.0
25 mg/kg TETA	266 ± 12.5	−11%	1.10 ± 0.70	×2.8
200 mg/kg TETA	284 ± 13.3	−5%	1.25 ± 0.59	×3.2
12 mg/kg bcTTM	303 ± 11.1	+2%	0.66 ± 0.19 ^c^	×1.7

**Table 2:** Comparison of the chelators TDMQ20, DPA, TETA, and TTM.

Entry		TDMQ20	DPA	TETA	TTM
***Coordination chemistry of the Cu–L complex***					
1	Cu selectivity	Yes	No	No	Yes
2	Structure of the complex	Discrete, L/M = 1/1	Versatile	Versatile	[Cu-Mo-S] clusters
***In vitro biochemistry***					
4	Inhibition Cu,Zn-SOD in vitro (IC_50_, µM)	~600	<100	~600	<0.2
5	ROS production in vitro (IC_50_, L/M = 1/1)	No	Yes (also with DPA/Cu = 2/1)	No	No
***Cu content in TX mouse and liver histology***					
6	Decrease in Cu in liver	Yes (25–50 mg/kg)	Yes (200 mg/kg) No (25 mg/kg)	No (≤ 200 mg/kg)	No (12 mg/kg)
7	Increase in Cu in urine	Yes (50 mg/kg) No (25 mg/kg)	Yes (200 mg/kg) Yes (≥ 25 mg/kg)	Yes (200 mg/kg) No (25 mg/kg)	No (12 mg/kg)
8	Decrease in Cu in kidney	Yes (25–50 mg/kg)	No (≤ 200 mg/kg)	No (≤ 200 mg/kg)	No (12 mg/kg)
9	Increase in Cu in feces	Yes (25–50 mg/kg)	No (200 mg/kg)	No (200 mg/kg)	Yes
10	Decrease in liver steatosis	Yes (complete at 50 mg/kg)	Yes (partial at 200 mg/kg)	nd ^a^	nd
***Protein content in TX mouse***					
11	Increase in serum holo-CP	Yes at 50 mg/kg, trend at 25 mg/kg	nd	nd	nd
12	Increase in ferroxidase activity of CP in the serum	Yes (25–50 mg/kg)	Yes (200 mg/kg)	nd	nd
13	Increase in liver CP	Yes (50 mg/kg)	Yes (200 mg/kg)	nd	nd
14	Increase in liver ATP7B	Yes (50 mg/kg)	Yes (200 mg/kg)	nd	nd
15	Normalization of CYP7A and CYP7B	Yes	nd	nd	nd

^a^ nd = not determined. For bibliographic references, see text.

## Data Availability

All data can be found either in this article or in the Supporting Information published alongside this article. No artificial intelligence tools were used (neither to design this research nor to execute, analyze, or write it).

## Supplementary Materials

The following supporting information can be downloaded at https://www.mdpi.com/article/10.3390/pharmaceutics17091237/s1, Tables S1–S7: Individual raw data for each mouse sample. Figure S1: Non-denatured Western blot analysis of serum ceruloplasmin: normalized apo-CP and holo-CP, respectively, with respect to albumin. Figure S2: Kinetics of ascorbate oxidation in the presence of TETA + Cu^2+^ or bcTTM + Cu^2+^.
