# Supplementary material for: TDMQ20 as A Drug Candidate for Wilson’s Disease: Comparison with D-Penicillamine, Trientine, and Tetrathiomolybdate In Vitro and In Mice
Source: Pharmaceutics. 2025 Sep 22;17(9):1237. doi: 10.3390/pharmaceutics17091237 (PMC12473319; doi:10.3390/pharmaceutics17091237)

Supporting Information

TDMQ20 as drug-candidate for Wilson's disease: comparison with D-penicillamine, trientine, and tetrathiomolybdate *in vitro* and in mice.

Table S1. Dosage of copper in mouse liver and urine. Individual raw data. and mean values ± SEM by group. n = 6 except otherwise indicated.

**Group**	**Cu concentration in liver** (mg/kg)						**Mean** (mg/kg)
Control	5.04	4.66	5.4	3.53	3.59	3.78	4.33
WD (untreated)	301	302	288	308	295	291	297.5
25 mg/kg TDMQ20	235	235	241	265	286	235	249.7
50 mg/kg TDMQ20	247	226	220	222	187	190	215.3
25 mg/kg DPA	261	289	210	297	280	-[a]	267.4
200 mg/kg DPA	237	244	230	273	256	281	253.5
25 mg/kg TETA	293	272	301	249	262	217	265.7
200 mg/kg TETA	316	273	332	270	255	256	283.7
12 mg/kg bcTTM	318	318	326	260	319	278	303.2

**Group**	**Cu concentration in urine** (mg/L)						**Mean** (mg/L)
Control	0.11	0.20	0.10	0.19	0.09	0.09	0.13
WD (untreated)	0.47	0.42	0.21	0.34	0.52	-[b]	0.39
25 mg/kg TDMQ20	0.30	0.37	0.30	0.94	0.39	-[b]	0.46
50 mg/kg TDMQ20	3.63	0.49	1.17	2.37	-[b]	-[b]	1.92
25 mg/kg DPA	1.44	3.95	1.09	1.83	0.81	-[b]	1.82
200 mg/kg DPA	2.75	3.87	4.09	6.40	-[b]	-[b]	4.28
25 mg/kg TETA	1.01	0.85	0.83	1.14	0.38	2.41	1.10
200 mg/kg TETA	1.14	1.95	1.73	1.55	0.64	0.49	1.25
12 mg/kg bc-TTM	0.51	0.74	0.95	0.47	0.63	-[b]	0.66

[a] Sample lost. [b] Fail to recover a sufficient volume.

Table S2. Dosage of copper in mouse kidneys. Individual raw data and mean values ± SEM by group. Outlier individual values in red have been removed from calculations. Values in blue stand for mean and SEM values calculated from n = 4/6 or 5/6 for the group of mice treated with TDMQ20 at 50 mg/kg or by DPA at 25 mg/kg, respectively.

**Kidney Cu**									
**Individual data**	**Control**	**WD**	**TDMQ20-25 mg/kg**	**TDMQ20-50 mg/kg**	**DPA-25 mg/kg**	**DPA-200 mg/kg**	**TETA-25 mg/kg**	**TETA-200 mg/kg**	**Bc-TTM-12 mg/kg**
	4.62	5.7	5.65	5.19	5.11	5.10	6.39	5.80	6.36
	4.83	5.76	4.64	5.11	7.00	7.66	6.60	4.29	6.42
	4.41	5.74	5.24	4.75	8.47	5.17	5.17	8.14	6.67
	4.19	6.45	4.19	4.25	7.08	7.22	5.38	7.58	5.49
	5.66	6.73	6.36	24.7	7.05	7.06	6.45	6.17	6.35
	4.26	7.3	4.43	21.2	4.24	7.37	6.13	6.21	4.99
	**Control**	**WD**	**TDMQ20-25 mg/kg**	**TDMQ20-50 mg/kg**	**DPA-25 mg/kg**	**DPA-200 mg/kg**	**TETA-25 mg/kg**	**TETA-200 mg/kg**	**Bc-TTM-12 mg/kg**
**Mean**	4.66	6.28	5.09	4.83	6.94	6.60	6.02	7365	6.05
**SEM**	0.22	0.27	0.34	0.21	0.54	0.47	0.25	0.56	0.27

Table S3. Proportions of apo-CP and holo-CP with respect to total CP in mouse serum. Individual raw data and mean values ± SEM by group. n = 3 for each group.

Group	**Percentage of apo-CP**					**Percentage of holo-CP**				
	**In each lane**			**Mean**	**SEM**	**In each lane**			**Mean**	**SEM**
**Control**	0.66	0.65	0.66	0.66	0.003	0.34	0.35	0.34	0.34	0.003
**WD**	0.85	0.83	0.86	0.85	0.009	0.15	0.17	0.14	0.15	0.009
**TDMQ20-25 mg/kg**	0.81	0.80	0.79	0.80	0.006	0.19	0.20	0.21	0.20	0.006
**TDMQ20-50 mg/kg**	0.77	0.75	0.76	0.76	0.006	0.23	0.25	0.24	0.24	0.006

Table S4. Apo-CP/albumin- and holo-CP/albumin ratios in mouse serum. Individual raw data and mean values ± SEM by group. n = 3 for each group.

Group	**apo-CP/albumin**			**Mean**	**SEM**
**Control**	0.62	0.93	0.99	0.85	0.11
**WD**	0.87	1.02	0.90	0.93	0.04
**TDMQ20-25 mg/kg**	1.00	0.90	0.75	0.88	0.07
**TDMQ20-50 mg/kg**	0.71	0.61	0.66	0.66	0.03

Group	**holo-CP/albumin**			**Mean**	**SEM**
**Control**	0.59	0.75	0.70	0.68	0.05
**WD**	0.16	0.23	0.20	0.20	0.02
**TDMQ20-25 mg/kg**	0.34	0.42	0.36	0.37	0.02
**TDMQ20-50 mg/kg**	0.46	0.49	0.46	0.47	0.01

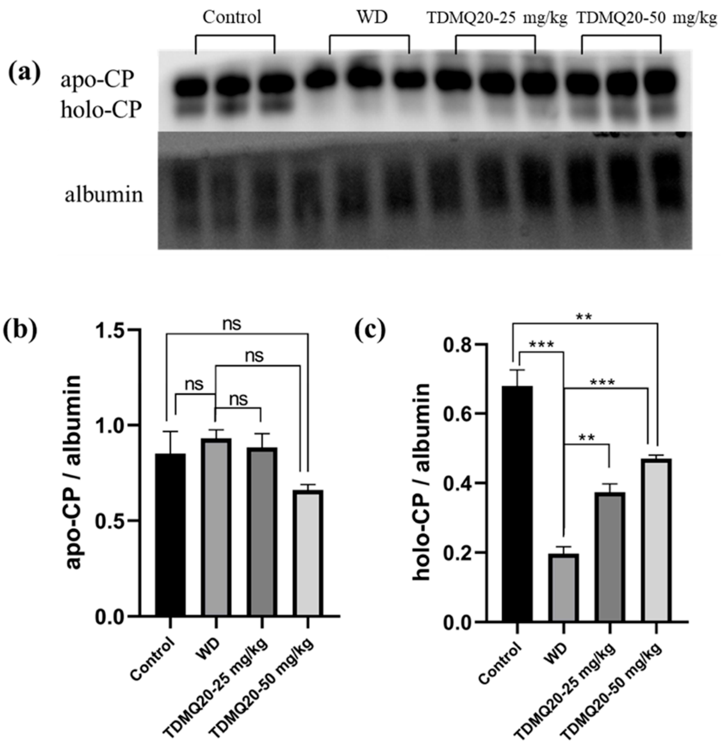

Figure S1. (a) Non-denaturated Western blot analysis of serum ceruloplasmin (CP, 30 μg of total proteins deposited in each lane). (b) and (c): Normalized apo-CP and holo-CP, respectively, with respect to albumin as internal standard; The results are the mean values ± SEM of 3 gel deposits for each group. Not significant (ns) when $p > 0.05$. The p values with statistical significance for holo-CP **(c)** are as follows: Control *vs*. WD ＜0.001, WD *vs*. TDMQ20-25 mg/kg = 0.002, WD *vs*. TDMQ20-50 mg/kg ＜0.001, Control *vs*. TDMQ20-50 mg/kg = 0.001.

Table S5. Dosage of ferroxidase activity of ceruloplasmin in mouse serum. Individual raw data and mean values ± SEM by group. n = 6 for each group.

**Ferroxidase activity of CP in serum (U/mL)**									
							**Mean**	**SEM**	
**Control**	11.28	11.65	11.64	11.36	11.67	11.48	11.51	0.07	
**WD**	7.28	6.04	6.64	5.35	7.65	5.86	6.47	0.36	-44% / control
**TDMQ20-12.5 mg/kg**	8.62	8.68	8.77	8.96	9.02	8.65	8.78	0.07	
**TDMQ20-25 mg/kg**	9.85	9.80	9.74	9.4	9.46	9.74	9.67	0.08	-16% / control
**TDMQ20-50 mg/kg**	12.26	12.15	12.58	13.00	12.83	12.2	12.50	0.15	+8% / control
**DPA-200 mg/kg**	10.86	10.9	10.48	10.83	9.91	10.3	10.55	0.16	-8% / control

Table S6. Dosage of the ratios ATP7B/β-actin and CP/β-actin in mouse liver. Individual raw data for each mouse liver, and mean values ± SEM by group. n = 3 for each group.

	**ATP7B/β-actin ratio in the liver**			**Mean**	**SEM**
**Control**	0.39	0.93	0.77	0.69	0.16
**WD**	0.14	0.12	0.15	0.14	0.01
**TDMQ20-25 mg/kg**	0.20	0.19	0.28	0.22	0.03
**TDMQ20-50 mg/kg**	0.43	0.40	0.39	0.41	0.01
**DPA-200 mg/kg**	0.47	0.43	0.38	0.43	0.03

	**CP/β-actin ratio in the liver**			**Mean**	**SEM**
**Control**	0.95	0.66	0.96	0.86	0.10
**WD**	0.62	0.37	0.54	0.51	0.07
**TDMQ20-12.5 mg/kg**	0.76	0.48	0.67	0.64	0.08
**TDMQ20-25 mg/kg**	0.63	0.67	0.64	0.65	0.01
**TDMQ20-50 mg/kg**	1.05	0.91	0.94	0.97	0.04
**DPA-200 mg/kg**	0.91	0.71	0.73	0.78	0.06

Table S7. Dosage of CYP7A1 and CYP7B1 in the mouse liver, by Tandem Mass Tag LC-MS/MS (arbitrary units). Individual raw data.

	**CYP7A1**			**CYP7B 1**		
**Control**	136.9	122.3	84.7	140.2	101.5	104
**WD**	59.4	75.3	75.7	180.2	143	126.2
**TDMQ20-25 mg/kg**	82.3	107.8	100.9	89.9	88.6	73.8
**TDMQ20-50 mg/kg**	132.7	109.4	112.5	48.9	53.8	49.8

Figure S2. Ascorbate oxidation measured by the decrease of ascorbate absorbance at 265 nm. in the presence of a) TETA/Cu^{2+} or b) bcTTM/Cu^{2+}. Drug/ Cu^{2+} molar ratio in the range 0-1.1. indicated on each trace.

(a) **(b)**

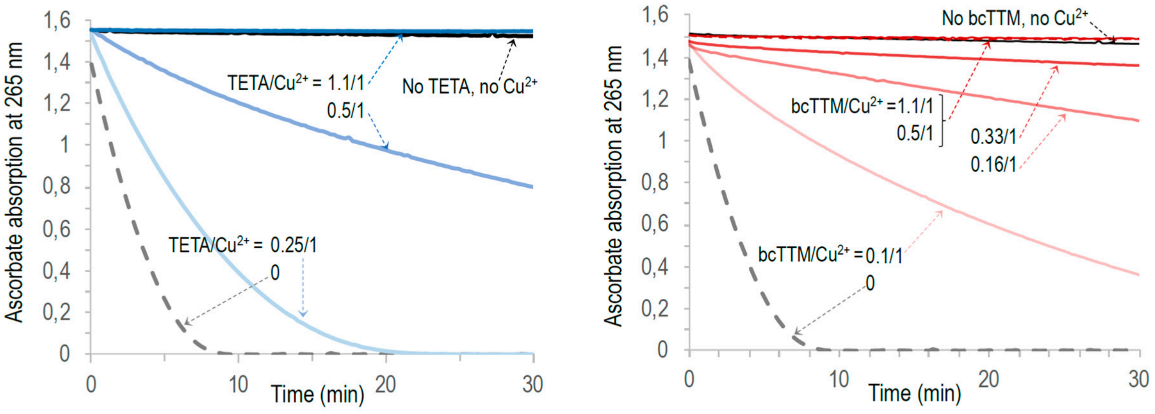

Supplement: Supplementary file 1 [file pharmaceutics-17-01237-s001.zip › pharmaceutics-3843637-supplementary.pdf]
